# Cognitive and emotional empathy after stimulation of brain mineralocorticoid and NMDA receptors in patients with major depression and healthy controls

**DOI:** 10.1038/s41386-020-0777-x

**Published:** 2020-07-28

**Authors:** Jan Nowacki, Katja Wingenfeld, Michael Kaczmarczyk, Woo Ri Chae, Ikram Abu-Tir, Christian Eric Deuter, Dominique Piber, Julian Hellmann-Regen, Christian Otte

**Affiliations:** Department of Psychiatry and Psychotherapy, Campus Benjamin Franklin, Charité – Universitätsmedizin Berlin, corporate member of Freie Universität Berlin, Humboldt-Universität zu Berlin, and Berlin Institute of Health, Hindenburgdamm 30, 12203 Berlin, Germany

**Keywords:** Endocrine system, Depression

## Abstract

Mineralocorticoid receptors (MR) are predominantly expressed in the hippocampus and prefrontal cortex. Both brain areas are associated with social cognition, which includes cognitive empathy (ability to understand others’ emotions) and emotional empathy (ability to empathize with another person). MR stimulation improves memory and executive functioning in patients with major depressive disorder (MDD) and healthy controls, and leads to glutamate-mediated N-methyl-D-aspartate receptor (NMDA-R) signaling. We examined whether the beneficial effects of MR stimulation can be extended to social cognition (empathy), and whether DCS would have additional beneficial effects. In this double-blind placebo-controlled single-dose study, we randomized 116 unmedicated MDD patients (mean age 34 years, 78% women) and 116 age-, sex-, and education years-matched healthy controls to four conditions: MR stimulation (fludrocortisone (0.4 mg) + placebo), NMDA-R stimulation (placebo + D-cycloserine (250 mg)), MR and NMDA-R stimulation (both drugs), or placebo. Cognitive and emotional empathy were assessed by the Multifaceted Empathy Test. The study was registered on clinicaltrials.gov (NCT03062150). MR stimulation increased cognitive empathy across groups, whereas NMDA-R stimulation decreased cognitive empathy in MDD patients only. Independent of receptor stimulation, cognitive empathy did not differ between groups. Emotional empathy was not affected by MR or NMDA-R stimulation. However, MDD patients showed decreased emotional empathy compared with controls but, according to exploratory analyses, only for positive emotions. We conclude that MR stimulation has beneficial effects on cognitive empathy in MDD patients and healthy controls, whereas NMDA-R stimulation decreased cognitive empathy in MDD patients. It appears that MR rather than NMDA-R are potential treatment targets to modulate cognitive empathy in MDD.

## Introduction

Patients with major depressive disorder (MDD) exhibit cognitive deficits in executive functioning, memory, and attention that may persist even after remission [1, 2]. Interestingly, there is evidence that MDD patients show altered concentrations of the steroid hormone cortisol that, in turn, is associated with deficits in these cognitive domains [3–5]. Yet, little is known about the role of cortisol in MDD patients in other cognitive domains that are also clinically relevant to MDD, such as social cognition—the process of identification, perception, and interpretation of social information [6].

Cortisol is released in response to stress and acts via glucocorticoid receptors (GR) and mineralocorticoid receptors (MR). GR are expressed across the brain, while MR are predominantly expressed in the hippocampus and prefrontal cortex [7–9]. Importantly, these brain areas are closely associated with processes of social cognition [10].

There is increasing evidence for an important role of the MR in cognitive processes in MDD patients and healthy individuals [5, 11]. In healthy humans, MR blockade impaired memory and executive functioning [12, 13], whereas MR stimulation by fludrocortisone improved memory processes [14, 15]. Because MR stimulation appears to improve cognitive processes in healthy individuals, it might serve as a potential treatment target to improve cognitive deficits in MDD patients. Indeed, we found in our own group that MR stimulation improved verbal memory, and executive functioning in MDD patients and healthy controls [16]. However, it remains open to question whether these beneficial effects of MR stimulation on cognition in MDD patients can be extended to social cognition.

Depressed patients often exhibit deficits in social cognition that contribute to impaired social functioning and quality of life [6]. One important aspect of social cognition is empathy that consists of a cognitive and an emotional component. While the former describes the ability to understand others’ emotions, the latter refers to the ability to feel with another person [17]. Overall, it appears, that several aspects of empathy are impaired in MDD patients [18]. However, the results are heterogeneous with studies showing decreased emotional empathy for positive emotions [19], increased emotional empathy for negative emotions [20], or no empathy differences compared with healthy controls (e.g., ref. [21]).

We previously found that modulating the MR affects social cognition (empathy) in health and disease. For instance, blockade of the MR reduced cognitive empathy in MDD patients [19]. Furthermore, MR stimulation enhanced emotional empathy in patients with borderline personality disorder and healthy controls [22]. Thus, MR stimulation appears to enhance empathy and these beneficial effects might be applicable to MDD patients also. Although our studies provide first evidence for beneficial effects of MR stimulation, the mechanisms of action by which fludrocortisone enhances social cognition (empathy) remain to be examined.

In reaction to stress, glucocorticoids activate the release of glutamate via the MR in the hippocampus and prefrontal cortex. Glutamate, in turn, binds upon the N-methyl-D-aspartate receptor (NMDA-R) [8, 23, 24], which is involved in several cognitive processes. For instance, knockout of the NMDA-R in the forebrain is associated with impaired social cognition in mice [25] and stimulation of the receptor by the agonist D-cycloserine (DCS) has beneficial effects on decision making, memory, and learning in healthy individuals [26, 27] and in a range of psychiatric populations [28]. Accordingly, the beneficial effects of MR stimulation on social cognition (empathy) might be enhanced by modulation of NMDA-R signaling.

In the current study, we examined whether the beneficial effects of MR stimulation by fludrocortisone can be extended to social cognition (empathy) in MDD patients. In addition, we examined whether these potential beneficial effects can be enhanced by coadministration of the partial NMDA-R agonist DCS. We hypothesized that (1) MR stimulation by fludrocortisone enhances cognitive and emotional empathy in MDD patients, and that (2) simultaneous NMDA-R stimulation by DCS additionally enhances the effects of MR stimulation on cognitive and emotional empathy.

## Materials and methods

### Participants

We examined 116 patients with MDD and 116 healthy controls. We recruited MDD patients from the Department of Psychiatry of the Charité—Universitätsmedizin Berlin (in- and outpatients), via our website and by means of flyers distributed in outpatient psychiatric and psychotherapy practices. We recruited healthy controls via our website, and by means of flyers that we distributed in universities and other public buildings. MDD patients and healthy controls were matched for age, sex, and years of education. All participants gave their written informed consent and received an expense allowance for participation. The study was approved by the local ethics committee (Landesamt für Gesundheit und Soziales Berlin, 16-0031-EK 11) and was conducted according to the Declaration of Helsinki. The study was registered on clinicaltrials.gov (NCT03062150).

We included participants in the age between 18–65 years. For MDD patients, additional inclusion criteria were the diagnosis of MDD according to the Diagnostic and Statistical Manual of Mental Disorders [DSM-5; ref. [29]] and a minimum score of 18 on the 17-item Hamilton rating scale for depression (HAMD) [30].

Exclusion criteria for all participants were: intake of any psychotropic medication within the past 5 days (except for antidepressant sleep medication and benzodiazepines on demand), substance abuse or dependency within the last half-year, diagnosis of schizoaffective disorder, bipolar disorder, or schizophrenia (healthy controls were free of any current, or past psychiatric disorder and psychotropic medication), pregnancy or lactation period, study medication intolerance, neuroendocrine disorders, organic brain disease (current or past), acute suicidality, endocrine disorders, neuroendocrine medication intake, abnormal cardiovascular conditions, or abnormal clinical laboratory.

### Procedure

All participants were pre-assessed for eligibility during a short telephone interview, except for inpatient MDD patients who were pre-assessed based on their medical record. Thereafter, participants were invited for an in-person visit for further diagnostic evaluation. During the in-person visit, participants gave their written informed consent, followed by a diagnostic clinical interview conducted by a trained and experienced physician or psychologist. The aim of the interview was to check inclusion and exclusion criteria, to diagnose or preclude any psychiatric disorder and to assess the general medical condition of the participants. For the diagnosis of psychiatric disorders, we conducted the Structured Clinical Interview for DSM-5 [31]. To measure the severity of the MDD, we used the HAMD [30] and the Beck Depression Inventory [BDI; ref. [32]]. To evaluate the general medical condition, we measured blood pressure and heart rate, we took blood samples for a safety laboratory, and we conducted an electrocardiography. All participants who were eligible for participation were invited for the testing day. The testing day was scheduled between 24 h and 1 week after the in-person visit.

Eligible participants were randomly assigned to one of four single-dose treatment conditions: (A) placebo + placebo, (B) fludrocortisone + placebo, (C) placebo + DCS, or (D) fludrocortisone + DCS. The Charité—Universitätsmedizin Berlin pharmacy conducted the randomization. We used a parallel group design with simple randomization: every four participants were randomized to one out of the four treatment conditions. Randomization was conducted separately for each group (stratified randomization) to ensure that 29 MDD patients and 29 healthy controls were randomized to each treatment condition.

The Charité—Universitätsmedizin Berlin pharmacy provided the medication to ensure blinding of participants and examiners. Participants received two identical-looking capsules that contained either fludrocortisone, DCS, or placebo. We used 0.4 mg fludrocortisone to stimulate the MR and 250 mg DCS to stimulate the NMDA-R. The dosage was based on studies that found cognitive enhancing effects in humans for one-time drug administration. For fludrocortisone, this was based on our own studies [15, 16, 33] and for DCS it was based on studies of other research groups [27, 34].

The testing day started for all participants at 1130 h. After a resting period of 30 min, participants received the first medication at 1200 h and the second medication at 1300 h. The Multifaceted Empathy Test (MET) was conducted between 1600 and 1700 h. This procedure was identical for all participants, in order to control for circadian rhythm of cortisol secretion [35]. Furthermore, by the afternoon cortisol concentrations have already much declined compared to peak levels after awakening [36] allowing agonistic effects at MR, which are largely but not fully occupied when cortisol levels are low [37].

We measured steroid hormone concentrations (cortisol, aldosterone, and DHEA-S), as well as blood pressure and heart rate every hour from 1200 to 1800 h. The effect of the treatment conditions on the cortisol response can be summarized as follows: cortisol concentrations decreased in both fludrocortisone conditions (fludrocortisone and fludrocortisone + DCS) across groups, whereas DCS had no effect on steroid hormone concentrations as described elsewhere [38].

### Multifaceted Empathy Test

We used a modified version [39] of the MET [40] to asses cognitive and emotional empathy. The computerized task consists of 30 pictures of people in emotional situations that are presented on a black screen. Pictures were presented in blocks of ten. In alternating order, participants were asked to rate ten pictures for cognitive empathy, and then ten pictures for emotional empathy. All blocks were presented twice, once for cognitive and once for emotional empathy. The order was pseudo-randomized, which implied that subsequent blocks presented different pictures. To measure cognitive empathy, participants were asked to indicate the emotion the person feels on the picture by choosing one out of four suggested emotions presented on the screen. The sum of all correct answers was calculated, leading to a minimum score of 0 and a maximum score of 30. To measure emotional empathy, participants were asked to indicate how much they empathize with the person on the picture on a Likert scale ranging from 1 (not at all) to 9 (very much). The sum score was calculated, leading to a minimum score for emotional empathy of 30 and a maximum score of 270. In addition, the mean score for positive and the mean score for negative emotions was calculated for cognitive and emotional empathy.

### Statistical analysis

For all statistical analyses, we used IBM SPSS Statistics (version 25). The analyses of the demographic information were conducted with *t*-tests for continuous data and chi-squared tests for categorical data.

The analyses of cognitive and emotional empathy were conducted with separate ANOVAs with the factors group (MDD patients vs. healthy controls), MR stimulation (fludrocortisone conditions vs. non-fludrocortisone conditions), and NMDA-R stimulation (DCS conditions vs. non-DCS conditions). Post hoc tests were Bonferroni corrected for multiple testing, and we used independent *t*-tests or paired sample *t*-tests, respectively.

We conducted several exploratory analyses: first, we analyzed whether cognitive and emotional empathy differed for positive and negative emotions. We used mixed ANOVAs with the between-subject factors group, MR stimulation, and NMDA-R stimulation and the within-subject factor valence (positive and negative emotions).

Second, within the group of female participants, we analyzed the effect of hormonal contraceptives with separate ANOVAs for cognitive and emotional empathy, and the factors hormonal contraception (intake vs. no intake of hormonal contraceptives), MR stimulation (fludrocortisone conditions vs. non-fludrocortisone conditions), and NMDA-R stimulation (DCS conditions vs. non-DCS conditions).

Third, we calculated change scores (delta) for cortisol, DHEA-S, and aldosterone by subtracting the mean of the two baseline values from the minimum post drug administration value for each hormone. Correlations between these delta values, and cognitive and emotional empathy for each treatment condition were calculated. Bonferroni corrections were applied to control for multiple testing.

Fourth, we calculated correlations between cognitive and emotional empathy, and depression severity (HAMD and BDI scores) within the group of MDD patients. Bonferroni corrections were applied to control for multiple testing.

The current study was powered based on the findings by Schultebraucks et al. [33], where we found an effect size of *d* = 0.70 for fludrocortisone vs. placebo on emotional dot probe in healthy young participants. Thus, we chose the emotional dot probe paradigm as primary outcome variable (unpublished data), and three other cognitive paradigms as secondary endpoints. The MET is one of the latter.

## Results

### Demographic information

MDD patients (*n* = 116) and healthy controls (*n* = 116) did not differ in age, sex, years of education, and in the proportion of hormonal contraception users among female participants. Fewer MDD patients were in a relationship than healthy controls. As expected, MDD patients had higher mean HAMD scores and higher mean BDI scores, as compared with healthy controls (see Table 1).

**Table 1:** Demographics characteristics.

	MDD	HC	Statistics
Age years	34.7 ± 13.3	34.9 ± 13.2	*t* (230) = 0.1, *p* = 0.90
Sex (women)	91 (78%)	91 (78%)	
Education duration	11.8 ± 1.3	12.1 ± 1.3	*t* (230) = 1.6, *p* = 0.12
In relationship (yes)	50 (43%)	67 (58%)	*χ*^2^(1) = 5.0, *p* < 0.05
Hormonal contraception	19 (21%)	19 (21%)	
HAMD	21.5 (3.4)	1.6 (1.3)	*t* (149) = −58.7, *p* < 0.001
BDI	25.7 (8.3)	1.4 (1.8)	*t* (125) = −30.8, *p* < 0.001

Demographic characteristics for depressed patients (MDD) and HC. Values represent mean ± SD or *n* (%).

*HAMD* Hamilton Rating Scale for Depression, *BDI* Beck Depression Inventory, *HC* healthy controls, *MDD* major depressive disorder.

### Cognitive empathy

**Fig. 1:**
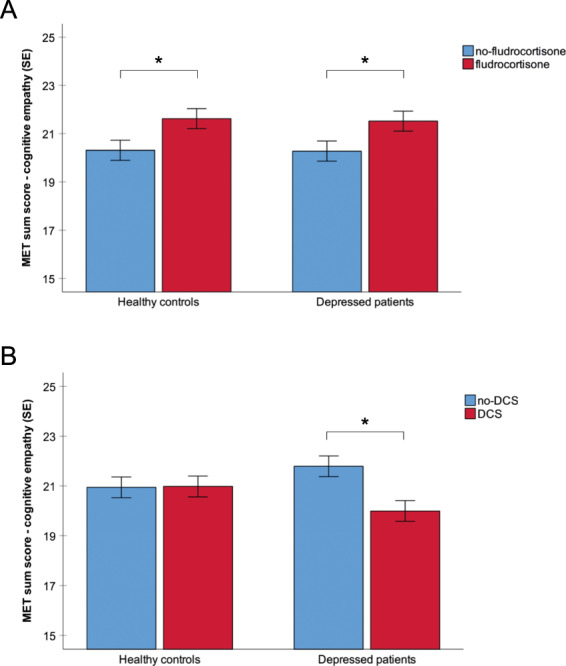
Cognitive empathy after MR and NMDA-R stimulation in patients with MDD and healthy controls. **a** Cognitive empathy scores were higher across groups after MR stimulation and **b** lower after NMDA-R stimulation in MDD patients. MET Multifaceted Empathy Test, error bars show standard error (SE), and significant differences are marked (*).

We found no main effect of group (*p* > 0.05), but a main effect of MR stimulation (*F*(1,224) = 9.4, *p* < .01, *η*^*2*^ = 0.04), indicating enhanced cognitive empathy after fludrocortisone administration (Fig. 1a). We also found a main effect of NMDA-R stimulation (*F*(1,224) = 4.5, *p* < 0.05, *η*^*2*^ = 0.02) and an interaction of group × NMDA-R stimulation (*F*(1,224) = 4.8, *p* < 0.05, *η*^*2*^ = 0.02). Post hoc tests indicated less cognitive empathy after DCS administration within the group of MDD patients only (*t*(114) = 2.8, *p* < 0.01; Fig. 1b). We found no interaction of MR stimulation × NMDA-R stimulation (*F*(1,224) = 0.5, *p* = 0.48, *η*^*2*^ = 0.002). Thus, combined stimulation of MR and NMDA-R had no effect on cognitive empathy across groups.

In exploratory analyses, we additionally found a main effect of valence (*F*(1,224) = 151.3, *p* < 0.001, *η*^*2*^ = 0.40), indicating higher scores of cognitive empathy for positive emotions compared with negative emotions across groups.

### Emotional empathy

**Fig. 2:**
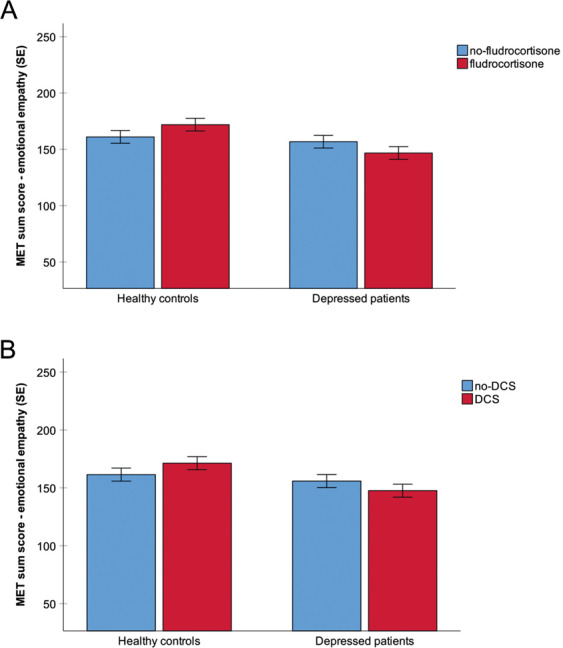
Emotional empathy after MR and NMDA-R stimulation in patients with MDD and healthy controls. MR stimulation (**a**) and NMDA-R stimulation (**b**) had no effect on emotional empathy scores in MDD patients and healthy controls. MET Multifaceted Empathy Test, error bars show standard error (SE).

We found a main effect of group (*F*(1,224) = 6.8, *p* < 0.05, *η*^*2*^ = 0.03), indicating less emotional empathy in MDD patients compared with healthy controls. We found no main effect of MR stimulation or NMDA-R stimulation (all *p*s > 0.05), and we found no interaction of MR stimulation × NMDA-R stimulation (*F*(1,224) = 0.2, *p* = 0.63, *η*^*2*^ = 0.001) or any other interaction (all *p*s > 0.05). Thus, separate and combined stimulation of MR and NMDA-R had no effect on emotional empathy across groups (Fig. 2).

**Fig. 3:**
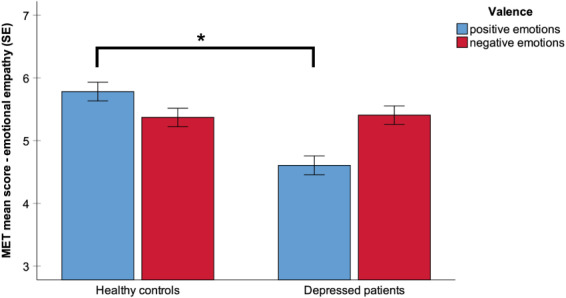
Emotional empathy for positive and negative emotions in patients with MDD and healthy controls. Emotional empathy scores were lower in MDD patients compared with healthy controls for positive emotions. MET Multifaceted Empathy Test, error bars show standard error (SE), and significant differences are marked (*).

In exploratory analyses, we additionally found a main effect of valence (*F*(1,224) = 4.1, *p* < 0.05, *η*^*2*^ = 0.02) and an interaction of group × valence (*F*(1,224) = 39.7, *p* < 0.001, *η*^*2*^ = 0.15). Post hoc tests revealed that MDD patients showed less emotional empathy compared with healthy controls for positive emotions only (*t*(224) = 5.5, *p* < 0.001; Fig. 3).

### Hormonal contraception

Exploratory analyses in women revealed no main effect of hormonal contraception, and no interaction between hormonal contraception × MR or × NMDA-R stimulation for cognitive and emotional empathy (all *p*s > 0.05). Thus, hormonal contraceptives had no effect, and did not influence the effect of MR or NMDA-R stimulation, on cognitive and emotional empathy in female participants.

### Correlational analyses

In exploratory analyses, we found no significant correlations between cognitive and emotional empathy and cortisol, DHEA-S, and aldosterone secretion within each treatment condition. Only 1 out of 24 correlations reached conventional significance (*p* < 0.05), and this result did not survive Bonferroni correction (*p* = 0.002, after 0.05/24).

In addition, we found no significant correlations between cognitive and emotional empathy, and depression severity in MDD patients. None of the four correlations survived Bonferroni corrections (*p* = 0.013, after 0.05/4) or reached conventional significance (*p* < 0.05).

## Discussion

We examined the separate and combined effect of MR and NMDA-R stimulation on cognitive and emotional empathy in MDD patients and healthy controls. Our main results are: (1) MR stimulation by fludrocortisone enhanced cognitive empathy in MDD patients and healthy controls, (2) NMDA-R stimulation by DCS decreased cognitive empathy in MDD patients, (3) cognitive empathy did not differ between MDD patients and healthy controls, and (4) emotional empathy was lower in MDD patients compared with healthy controls, but according to exploratory analyses, only for positive emotions.

Our results partly confirm our first hypothesis that MR stimulation by fludrocortisone enhances cognitive and emotional empathy in MDD patients. We found that MR stimulation enhanced cognitive, but not emotional empathy in MDD patients and healthy controls. This is partly in line with prior research of our group [19, 22]. We previously showed that MR blockade decreased cognitive empathy in MDD patients to the level of healthy controls [19], and that MR stimulation enhanced emotional empathy in patients with borderline personality disorder and healthy controls [22]. Together, our studies provide strong evidence for the involvement of the MR in social cognition (empathy) in health and disease. The current research shows, in addition, that the beneficial effects of MR stimulation on executive functioning and memory in MDD patients [16] can be extended to social cognition (empathy), another clinically relevant cognitive domain in MDD [6].

We could not confirm our second hypothesis that simultaneous MR and NMDA-R stimulation enhances the effects of single MR stimulation on cognitive and emotional empathy in MDD patients. In contrast, stimulation of the NMDA-R decreased cognitive empathy in MDD patients, but not in healthy controls and the combined stimulation of both receptors showed no (additive) effect. We assumed synergistic effects of the MR agonist fludrocortisone and the partial NMDA-R agonist DCS on empathy, based on findings that (1) MR stimulation leads to glutamatergic NMDA-R activation [8, 41], and (2) that MR agonism with fludrocortisone [15, 16, 33] and partial NMDA-R agonism with DCS [27, 34] have cognitive enhancing effects. However, the use of the partial NMDA-R agonist DCS does not allow examination of whether potential beneficial effects of MR stimulation depend on the NMDA-R. This would rather require NMDA-R blockage with an antagonist parallel to MR stimulation. Interestingly, the NMDA-R antagonist memantine improved memory by inducing neurogenesis in mice [42], and reversed the adverse effects of long-term glucocorticoid administration on hippocampus volume in humans over a period of several months [43].

The following should be considered when interpreting our results. First, the MR agonist fludrocortisone inhibits the hypothalamic–pituitary–adrenal axis leading to decreased secretion of cortisol [44, 45]. Given the higher binding affinity of cortisol for MR than GR, the decrease in cortisol concentrations is accompanied by lower GR occupation relative to MR occupation [46]. Thus, we examined the interplay between MR and GR-mediated effects rather than isolated effects of MR stimulation on empathy. Second, in the current study, we examined empathy 4 h after MR stimulation by fludrocortisone. Within this timeframe, late genomic MR and GR-mediated effects rather than early nongenomic actions occur [7]. Animal studies have shown rapid effects of glucocorticoids on glutamate transmission in the hippocampus, which were mediated by MR [8, 41]. It is thus possible that in humans, MR stimulation by fludrocortisone exerts early effects on glutamate transmission. Future studies should examine this question.

In terms of mechanisms, studies have shown that cognitive empathy processes are associated with the prefrontal cortex and emotional empathy processes are linked to the hippocampus, amygdala, and hypothalamus [47]. Interestingly, in MDD patients decreased MR expression has been found in the prefrontal cortex and hippocampus [48–50], and for the NMDA-R studies showed receptor downregulations in the prefrontal cortex and upregulations in the amygdala [51]. These findings fit very well with our finding that MR and NMDA-R stimulation changes cognitive empathy in MDD patients. Several other lines of evidence suggest an important role of the MR and NMDA-R in MDD. For instance, MR stimulation by fludrocortisone improved antidepressant treatment [52], and NMDA-R stimulation by DCS showed antidepressant effects [53]. Our research adds to the literature, showing that both receptors shape processes of social cognition (empathy) in MDD patients.

Our analyses of emotional valence indicated that independent of receptor stimulation, MDD patients showed less emotional empathy for positive emotions than healthy controls. The finding fits very well with studies showing that MDD patients suffer from impaired processing of positive emotions (e.g., refs. [54–56]). Furthermore, we replicated and extended an earlier study by our group [19] in a larger and younger sample of MDD patients. However, we did not replicate our earlier finding of increased cognitive empathy in MDD patients compared with healthy controls. Several other studies found no difference in cognitive empathy, but a bias toward negative emotional stimuli in MDD patients compared with healthy controls [20, 57]. Overall, the results suggest that MDD patients suffer from a mood congruent bias in emotional empathy processes that Beck [58] described in the cognitive model of depression (see also refs. [10, 54, 59]).

Our study had several strengths. First, we examined a comparatively large sample of unmedicated MDD patients. Rütgen et al. [60] argued that most research in the field had examined medicated patients, which restricts generalizability and that might have contributed to the heterogeneity of results in the field [18]. Importantly, one small longitudinal study showed that antidepressants influenced empathy in MDD patients [60]. Second, we carefully matched MDD patients and healthy controls according to age, sex, and education years. Third, there was an equal number of women using oral contraceptives in both groups. Although oral contraceptives have been shown to impact on cognitive empathy, affective responsiveness, and perception of emotional valence [61–63], in the current study we did not find an effect of oral contraceptives on cognitive and emotional empathy.

We would also like to acknowledge some limitations. Our study used the MET as a single empathy measurement instrument, which restricts generalizability of our findings. Several studies that used multiple empathy measurement instruments in the same sample found group differences for some, but not all empathy paradigms [19, 64, 65]. A related limitation is that the MET only assess state empathy. Other measurement instruments assess trait empathy, such as the Interpersonal Reactivity Index [66]. Recently, Banzhaf et al. [57] showed that empathy alterations in MDD patients are different for state and trait empathy. Future studies should therefore use several empathy measurements to ensure a widespread assessment of the concept of empathy. However, this needs to be weighed against the problem of multiple testing associated with several outcome variables.

Overall, our research shows that the beneficial effects of MR stimulation by fludrocortisone on several cognitive domains can be extended to aspects of social cognition, i.e., cognitive empathy in MDD patients and healthy controls. It appears that MR rather than NMDA-R are potential treatment targets to modulate cognitive empathy in MDD.

## Funding and disclosures

This study was funded by a grant from the German Research Foundation (Deutsche Forschungsgemeinschaft, DFG; OT 209/7–3) to C.O. and K.W. M.K. is a participant in the BIH-Charité Clinician Scientist Program funded by the Charité—Universitätsmedizin Berlin and the Berlin Institute of Health. C.O. has received honoraria for lectures and/or scientific advice from Allergan, Ferring, Fortbildungskolleg, Limes Klinikgruppe, Lundbeck, MedOnline, Medical Tribune, Neuraxpharm, SAGE Therapeutics, and Stillachhaus. All other authors reported no potential conflicts of interest. The study was conducted in cooperation with NeuroCure – Cluster of Excellence at Charité – Universitätsmedizin Berlin. Open access funding provided by Projekt DEAL.

## Supplementary information

Consort flowchart
