# Supplementary material for: Cognitive and emotional empathy after stimulation of brain mineralocorticoid and NMDA receptors in patients with major depression and healthy controls
Source: Neuropsychopharmacology. 2020 Jul 28;45(13):2155–61. doi: 10.1038/s41386-020-0777-x (PMC7785026; doi:10.1038/s41386-020-0777-x)
Supplement: Supplementary file 1 — Consort flowchart [file 41386_2020_777_MOESM1_ESM.docx]

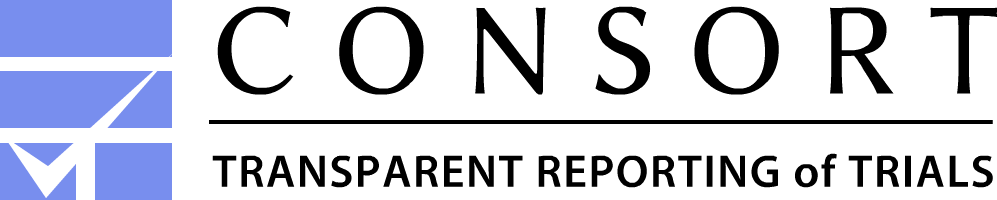


**CONSORT 2010 Flow Diagram**

**Healthy controls**

Allocated to intervention (n=116)

♦ Received allocated intervention (n=116)

♦ Did not receive allocated intervention (give reasons) (n=0 )

**Depressed patients**

Allocated to intervention (n=116)

♦ Received allocated intervention (n=116)

♦ Did not receive allocated intervention (give reasons) (n=0 )

## Follow-Up

Analysed (n=116 )
♦ Excluded from analysis (give reasons) (n=0 )

## Analysis

Analysed (n=116 )
♦ Excluded from analysis (give reasons) (n=0 )

Lost to follow-up (give reasons) (n=0 )

Discontinued intervention (give reasons) (n=0 )

Lost to follow-up (give reasons) (n=0 )

Discontinued intervention (give reasons) (n=0 )

## Enrollment

## Allocation

Randomized (n= 232 )

Excluded (n=25 )

♦  Not meeting inclusion criteria (n= 24)

♦  Declined to participate (n= 1 )

♦  Other reasons (n= 0 )

Assessed for eligibility (n=257 )
